# An Alpha and Theta Intensive and Short Neurofeedback Protocol for Healthy Aging Working-Memory Training

**DOI:** 10.3389/fnagi.2016.00157

**Published:** 2016-07-07

**Authors:** Joana Reis, Ana Maria Portugal, Luís Fernandes, Nuno Afonso, Mariana Pereira, Nuno Sousa, Nuno S. Dias

**Affiliations:** ^1^Life and Health Sciences Research Institute (ICVS), School of Health Sciences, University of MinhoBraga, Portugal; ^2^ICVS/3B’s – PT Government Associate LaboratoryGuimarães, Portugal; ^3^Clinical Academic Center – BragaBraga, Portugal; ^4^Digital Games Research Center, Polytechnic Institute of Cavado and AveBarcelos, Portugal

**Keywords:** EEG, neurofeedback, healthy aging, cognitive training, working memory, alpha, theta

## Abstract

The present study tested the effects of an intensive and short alpha and theta neurofeedback (NF) protocol in working memory (WM) performance in a healthy elder population and explored the effects of a multimodal approach, by supplementing NF with cognitive tasks. Participants were allocated to four groups: NF (*N* = 9); neurofeedback supplemented with cognitive training (NFCT) (*N* = 8); cognitive training (CT) (*N* = 7) and sham neurofeedback (Sham-NF) (*N* = 6). The intervention consisted in 30-min sessions for 8 days. The NF group presented post intervention increases of alpha and theta relative power as well as performance in the matrix rotation task. In addition, a successful up training of frontal theta showed positive correlation with an improvement of post-training alpha and a better performance in the matrix rotation task. The results presented herein suggest that an intensive and short NF protocol enables elders to learn alpha and theta self-modulation and already presents moderate improvements in cognition and basal EEG. Also, CT group showed moderate performance gains on the cognitive tasks used during the training sessions but no clear improvements on neurophysiology and behavioral measurements were observed. This study represents a first attempt to study the effects of an intensive and short NF protocol in WM performance of elders. The evidence presented here suggests that an intensive and short NF intervention could be a valid alternative for introduction of older populations to NF methodologies.

## Introduction

Modern societies have been witnessing a significant increase in life expectancy. Therefore, an increase in the burden of age-related conditions has been observed, namely in cognitive performance. The most documented cognitive changes in the aging brain are slower processing speed (Salthouse, 1996), poor encoding of information into episodic memory (Balota et al., 2000) and a deficit in inhibitory processing (Kramer et al., 1994). Also a considerable decline in executive functions such as working memory (WM) (Grady, 2000), attention (Madden, 1990; Connelly et al., 1991) and cognitive flexibility (Cepeda et al., 2001) were documented. WM is described as the ability of short-term retention of information, while allowing it to be prioritized, modified, utilized and protected from interference. WM is an essential feature in human cognition and it is typically reduced in older adults.

The most prevalent rhythm in the adult electroencephalogram (EEG) is the alpha (8-12 Hz). Alpha oscillations are related to the psychological state and cognitive performance of the subjects (Adrian and Matthews, 1934; Klimesch, 1997, 1999). In frontal sites, alpha activity might be caused by thalamic and anterior cingulate cortex activity, which addresses attention and WM processing. Another important EEG rhythm is the theta (4–8 Hz) and its activity is related to cognitive performance, especially during memory tasks. Lower theta activity is related to resting state, unless in sleepy stages (Vaitl et al., 2005), and enhanced activity is related to memory encoding and retrieval (Jacobs et al., 2006; Cavanagh et al., 2012; Itthipuripat et al., 2013), proving its relationship with hippocampus functioning (Cantero et al., 2003).

Neurology and electrophysiology studies found differences in the brain through aging. Indeed, age-related poorer cognitive performance might be paired with some registered EEG differences, as a slower general EEG activity (Giaquinto and Nolfe, 1986; Breslau et al., 1989; Prichep, 2007), an enhanced parietal and temporal delta (Breslau et al., 1989), and changes in coherence (Prichep, 2007) mainly between frontal and parietal structures that share attentional and information processing pathways. In a study performed by Dias et al. (2015) EEG spectral power and coherence were related to age-related performance decline on the Wisconsin Card Sorting Test (WCST) (Dias et al., 2015). Hence, the relationship between cognitive performance and EEG signals suggest that EEG features in specific cortical sites may be used as targets of neuromodulation strategies.

Neurofeedback (NF) is a brainwave training technique that has been used to enhance performance in athletes and musicians, creativity, attention and WM (Gruzelier, 2014b). It has also been used in several clinical conditions such as attention deficit hyperactive disorder (ADHD) (Heinrich et al., 2007), autism spectrum disorder (Coben et al., 2010), depression (Hammond, 2005) and epilepsy (Egner and Sterman, 2006). NF acts by giving feedback to the subject about his electrophysiological state and directing it to the desired activity (Vernon, 2005; Hammond, 2011). The focus on NF as a tool to increase physical and cognitive performance (commonly referred as peak performance training) is growing. Most studies focus on alpha and theta training in healthy adult populations, stressing its effects in attention and WM improvements (Angelakis et al., 2007; Lecomte, 2011). In terms of protocol duration, greater results are observed in longer NF protocols with 10 sessions on average (Lecomte, 2011) and with resting days between sessions. Studies showed that in a healthy population upper-alpha training had positive effects on mental rotation task performance (Bauer, 1976; Hanslmayr et al., 2005; Zoefel et al., 2011). Also, other study reported better cognitive performance in a WM task in the participants who were able to modulate upper-alpha in a NF up-training protocol (Escolano et al., 2011).

The studies dedicated to NF intervention in elderly populations for preservation of cognitive functions are still sparse, with very small samples, distinct protocols and provide conflicting evidence (Angelakis et al., 2004; Babiloni et al., 2007; Lecomte, 2011; Becerra et al., 2012; Wang and Hsieh, 2013; Gruzelier, 2014a; Staufenbiel et al., 2014). Importantly, however, these studies revealed that NF learning could be successfully applied to aged individuals. Following such evidence, alongside with other studies that linked alpha rhythm to attention processes and theta to WM performance, herein we studied the effects of an 8-days combined alpha and theta intensive NF training in EEG modulation and cognitive performance in older adults, especially in WM tasks. Considering the importance traditionally attributed to cognitive training on clinical settings (Lustig et al., 2009), as well as the advantages generally highlighted on multimodal approaches of cognitive enhancement, the combination of traditional cognitive tasks and NF methodologies seem to afford the advantage of supplying the subject with two types of performance assessment. Thus, besides a sham-NF group, two extra groups were additionally included: an experimental group that supplements NF with traditional cognitive tasks; and cognitive training group controlling for the effects of cognitive tasks alone. In order to assess the results of all training methodologies, EEG and cognitive evaluations were done before and after training. Cognitive performance during training and its translation to attention, resources mobilization and working-memory gains were the main outcomes evaluated on each training approach.

## Methodology

### Participants Recruitment and Characterization

For this study, 34 right-handed healthy participants (16 males and 18 females) aged above 55 years (mean age of 65.97 ± 6.63 years), were recruited from a Health Care Centre from Braga, Portugal. Only participants without any diagnosed dementia, cerebrovascular or neurological pathology were invited to take part in the study. All participants were asked about their educational background, current or previous occupations as well as prescribed medication. The participants did not present a high academic level (mean years of schooling: 6.69 years ± 3.71 years) and were mostly retired. The cohort was established in accordance with the principles expressed in the Declaration of Helsinki and the work was approved by the national ethical committee and by local ethics review boards. (Ethics Subcommittee for Life and Health Sciences of the University of Minho Ethics Committee of Hospital de Braga, Portugal). All the participants sign a voluntarily informed consent for the use of the collected data.

Initially the participant’s neuropsychological profile was assessed by a team of trained psychologists, using a battery of cognitive tests to measure cognitive state and psychological tests to assess the presence of depressive symptoms. Geriatric Depression Scale (GDS), Montreal Cognitive Assessment (MoCA) and the Mini-Mental State Examination (MMSE) were comprised in this battery. At the end of the study, all participants completed a questionnaire about their general opinion of the study.

### EEG Acquisitions

All EEG signals were acquired with the QuickAmp^®^, Brain Products, GmbH or the ActiCHamp^®^, Brain Products, GmbH. Both systems use the international 10–20 system with 32-channels standard electrode layout with ground and reference electrodes. The whole system was constituted by: Ag/AgCl active electrodes, a cap – actiCAP or EASYCAP (Brain Products, GmbH) – electrolyte gel and straps to keep the cap in place. Ground was located at forehead and reference was FCz channel when using QuickAmp equipment and Cz when using the ActiCHamp equipment. For each participant, the same equipment was used through all the sessions.

### Experimental Design

**FIGURE 1:**
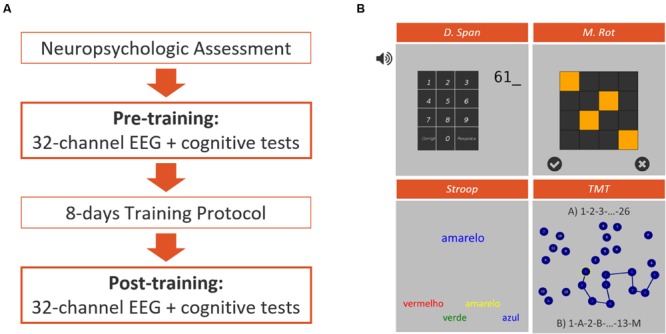
Experimental design **(A)** representation of the different phases of the intervention protocol featuring a traditional neuropsychological assessment, a pre- and post-training sessions for EEG assessment simultaneously to the performance of a computerized test battery, and 8 days of intensive training. **(B)** Representative screenshots of the four computerized tests applied on pre- and post-training assessment sessions, featuring the Auditory Backward Digit Span Test (D. Span), the Matrix Rotation Test (M. Rot), the Stroop Test and the Trail-Making Test (TMT).

All the participants followed a 12-day protocol accordingly to the diagram in **Figure 1**. During the intervention, participants were sited in an illuminated and acclimatized room, distancing 50–80 cm from a 17-inch computer screen with touch technology. All the stages of the study were conducted in Hospital of Braga.

**FIGURE 2:**
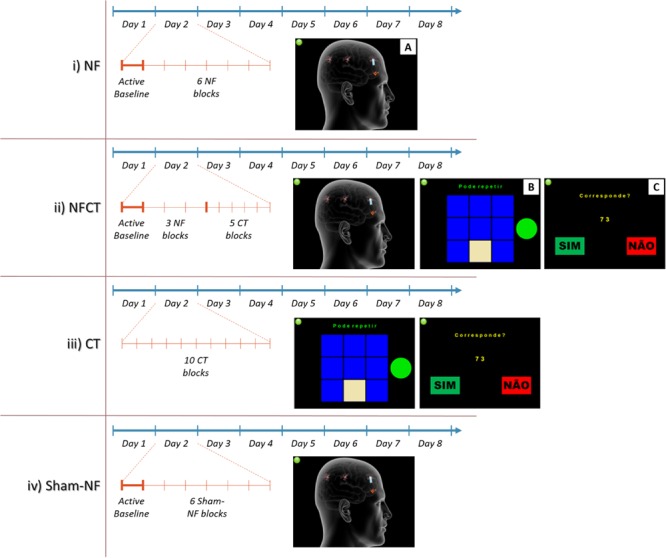
**Representation of the implemented 8-days training intensive and short NF protocol featuring four groups: NF, NFCT, CT and Sham-NF.** The NF group (i) performed 4 days of alpha and 4 days of theta training. Each day involved an active baseline followed by six blocks of NF **(A)**. The NFCT group (ii) performed NF supplemented with cognitive tasks [Corsi Block-Tapping Task **(B)** and n-Back Task **(C)**]. Each day consisted in an active baseline followed by 3 blocks of NF (similar to **A**) and 5 blocks of NC tasks. The CT group (iii) performed, each day, 10 blocks of the abovementioned NC tasks. The Sham-NF group (iv) followed the same protocol structure as applied to the NF group.

Participants were randomly allocated to four experimental groups according to the diagram presented in **Figure 2**. From the initial 34 participants, 1 dropped out and 3 outliers were excluded from the analysis which results in a final *N* = 30 participants. The participants were divided in the following groups; (i) NF (*N* = 9); (ii) NF supplemented with cognitive training (NFCT, *N* = 8), (cognitive training consisting of four different tasks: Corsi-Block Tapping Task – Forward and Backward; and n-Back Task– 1-Back and 2-Back); (iii) cognitive training alone (CT, *N* = 7) and (iv) sham neurofeedback (sham-NF, *N* = 6).

During the sessions, EEG signals were acquired continuously using the BCI++ platform (Perego et al., 2009), sampled at 500 Hz from the Fp1, Fp2, Fz and Pz channels. The NF and sham-NF protocols were divided in 6 5-min blocks and preceded by a 3-min active baseline. The NFCT protocol consisted in 3 5-min NF blocks (also preceded by a 3-min active baseline) and 5 3-min blocks of the above-described cognitive tasks (each task was headed by a 1-min eyes open baseline). The CT protocol only consisted of 10 3-min blocks of the cognitive tasks (each one also headed by a baseline). The training sessions had a duration of 30-min for all experimental groups.

In order to guarantee that all participants presented a minimum attention level to perform the tasks, in the first 2 days they were submitted to a modified version of the Arrow Flanker Test adapted from the Psychology Experiment Building Language (PEBL; Mueller and Piper, 2014). All participants scored above 93.5% accuracy in the second test day and as a result, no subject was excluded from the study based on attention deficits.

After all the training sessions were completed a questionnaire regarding the participant’s general opinion about the study was applied. On a scale of 1 to 4 (being 3 “like” and 4 “like very much” to participate on the study) all participants answered with 3 or 4.

#### Participants Pre- and Post-Training Evaluations

All participants were characterized before and after the training protocol. At the same time, 32-channel EEG signals were acquired while participants performed 4 modified computerized cognitive tests: Stroop Test, Matrix Rotation Test, Trail-Making Test and Auditory Backward Digit Span Test, also adapted from PEBL (Mueller and Piper, 2014). The EEG signals acquired during the cognitive tests were synchronized with PEBL using OpenVibe software (Renard et al., 2010). All cognitive tests were preceded by a 1-min eyes-open baseline, where the participants were instructed to relax and minimize blinking and body movements while staring at the center of a gray computer screen.

To study the training effects on WM we used a measure of the mean accuracy for the Matrix Rotation and a combined measure of memory span and number of correct trials for the Digit Span. Differences between the pre- and post-training were calculated to evaluate the individual alterations induced by the different intervention protocols.

#### Neurofeedback

The NF task used in this study was fully designed and implemented by our team using BCI++ (Perego et al., 2009), a platform for custom development of C++ and Matlab^®^ (Mathworks, Natick, MA, USA) based game paradigms and processing algorithms.

##### Online signal processing

All NF training sessions were preceded by a 3-min active baseline. In this baseline, the power spectrum density (PSD) average was calculated for both alpha and theta rhythms. Alpha band was adjusted to the individual alpha peak frequency (IAPF) and set as IAPF ± 2 Hz. Theta was set from 4Hz to IAPF-3 Hz. The active baseline PSD was used as a participant-specific reference for the following NF training session and updated for each day of training. During the NF training, PSD was calculated online in 1 s windows and updated to the participant every 200 ms. Feedback was calculated as the ratio of the PSD calculated at Fz to the baseline PSD.

Artifact contaminated signals influence the NF learning outcome since their power affects frequency bands often used for NF training. Thus, data windows showing contamination of ocular artifacts, like eye blinks and saccades, were detected online and discarded from feedback whenever the signal amplitude of the Fp1 (vertical eye movements) or Fp2–Fp1 (horizontal eye movements) exceeded an adjustable threshold (the values ranged from 60 to 100 μV). In this case, the feedback was suppressed and the length of the bar did not change.

##### Neurofeedback paradigm

The NF virtual scenario consisted in the representation of a human head, three neurons and a flame, as depicted in **Figure 2A**. The feedback was given through a blue bar (symbolizing water) coming out of the neuron that must be increased by the participant in order to reach the flame. The blue bar length mirrored the aforementioned PSD ratio. Three neuronal cells indicated the three levels of the game (i.e., easy, medium and hard). The maximum length of the bar corresponded to 95% of the maximum amplitude measured during the active baseline, in a specific rhythm.

To assure comparability, the active baseline and the training use the same virtual scenario. During the baseline, the blue bar was locked to the first level and its length was randomly assigned. The subject was asked to count the number of times the bar reached the flame in order to maintain an active mental state (Zoefel et al., 2011; Enriquez-Geppert et al., 2014).

#### Cognitive Training Tasks

For the cognitive training, two WM cognitive tasks were implemented as illustrated on **Figure 2B**, the Corsi Block-Tapping Task, adapted from PEBL, and on **Figure 2C** the n-Back Task. In the Corsi Block-Tapping Task a sequence of up to nine identical spatially separated blocks is highlighted and the participant has to reproduce it, in either forward or backward order. If the sequence is reproduced correctly the next sequence is increased by one block, if not, it is decrease by one block. Each block has 15 trials.

In the n-Back Task the participant is continuously presented with digits and has to indicate whether or not the current digit matches the one n instance before (1-back – 1 instance before; 2-back – 2 instances before). Each block has 65 trials.

### Off-Line Signal Processing

All EEG data collected was processed off-line using EEGLAB, a MATLAB toolbox (Delorme and Makeig, 2004). Firstly, EEG signals acquired during the pre- and post-training evaluation sessions were filtered using high-pass (>0.2 Hz) and low-pass (<35 Hz) filters. To filter the signals, we used EEGLAB function *eegfilt* that implements a two-way least-squares FIR (finite-duration impulse response) digital filter with order calculated by default as 3^∗^(sample rate/low cut off frequency). Channels with contaminated or compromised data due to physiological (e.g., muscle activity, ECG, respiratory and skin artifacts) and extra physiological artifacts (e.g., interference originated from other equipment, alternating current or the electrodes) were then discarded and thereafter interpolated from neighboring channels. Then ocular artifacts were detected and removed using an algorithm based on independent component analysis (ICA; Makeig et al., 1996). The signal was segmented in 1-s windows both for baseline and activity records. The segments that contained artifacts were rejected from further analysis. Values above 50 μV and below -50 μV were marked as artifacts and segments with a difference between the lowest and highest amplitudes higher than 60 μV were also considered artifacts. Channels were grouped in four pools, frontal region on left hemisphere (FL), frontal region on right hemisphere (FR), parietal region on left hemisphere (PL) and parietal region on right hemisphere (PR). The average of three channels per area were considered for analyses (FL pool: FC5, F3 and FC1 electrodes; FR pool: FC6, F4 and FC2 electrodes; PL pool: CP5, P3 and CP1 electrodes; PR pool: CP6, P4 and CP2 electrodes. The PSD and coherence between signal pairs were calculated for each segment in both baseline and activity periods. PSD was calculated for Fz channel and for the four pools of channels. Coherence was calculated between the pools FR-FL, FR-PL, FR-PR, FL-PL, FL-PR, and PR-PL. Alpha and theta bands were adjusted according to the participant’s alpha peak frequency using the same methodology applied in training sessions. Relative PSD were also calculated based on the ratio of the mean PSD of each frequency band to the broadband (i.e., 0.2 – 35 Hz) PSD.

In order to assess the subject ability to modulate EEG rhythms, alpha and theta PSD were calculated for each day of training. Because only 4 EEG channels (i.e., Fp1, Fp2, Fz and Pz) were acquired during training, the ICA-based approach to ocular artifacts correction was not an option. The artifact rejection and segmentation was performed as explained for the EEG signals acquired on pre- and post-training. In each day, the PSD mean of the four best blocks (blocks in which the participants could reach a higher alpha or theta power) was calculated. The mean value of baseline PSD for the 8 days of training was subtracted to the block-wise PSD values. Alpha and theta PSD gradients were extracted from the linear regression of the baseline-corrected PSD values for the 4 days of training. Following the same approach, relative PSD differences were also calculated based on the ratio of the mean PSD on each band to the broadband (i.e., 0.2 – 35 Hz) PSD. Relative PSD gradients were calculated likewise.

The performance measures in the cognitive training were also assessed. A combined measure between the span and number of correct answers (score) in Corsi-Block tapping task and the percentage of correct answers in n-Back task (i.e., accuracy), were extracted for the 8 days of training of participants from groups NFCT and CT. A linear regression of the performance measures daily series was extracted similarly to NF linear regressions in order to obtain the performance gradients.

### Statistical Analysis

Non-parametric tests were used for all statistical analyses. For comparisons between groups the Kruskal–Wallis ANOVA was used. For testing for positive or negative effects of the intervention (testing if the median is greater or smaller than 0) the one-sample Wilcoxon signed-rank test was used. Kendall’s Rank Correlation Coefficient Test was performed to observe statistical dependence between measures. All statistical analyses were performed using OriginLab^®^ (OriginLab, Northampton, MA, USA) and significance was considered for *p*-values below 0.05.

## Results

**Table 1** shows the positive correlations found between the traditional neuropsychological tests (MMSE, MOCA, D.Direct, FAS, Codif., and CLOX) and the computerized versions of Digit Span and Matrix Rotation Tests. The other two tests (Trail-Making Test and Stroop Test) did not correlate to any measures of the traditional neuropsychological battery.

**Table 1:** Kendall correlation coefficients between digit span score and matrix rotation accuracy in the pre-training cognitive battery and various measures of the neuropsychological assessment implemented at the start of the protocol (*N* = 34).

	MMSE	MOCA	D. Direct	FAS (Ad.)	Codif.	CLOX
D. Span Score (Pre)	0.38^∗∗^	0.27^∗^	0.47^∗∗∗^	0.34^∗∗^	0.31^∗^	0.33^∗^
M. Rot Acc. (Pre)	0.46^∗∗∗^	0.50^∗∗∗^	0.48^∗∗∗^	0.55^∗∗∗^	0.40^∗∗^	0.32^∗^

*^∗^*p* < 0.05, ^∗∗^*p* < 0.01, ^∗∗∗^*p* < 0.001.*

**FIGURE 3:**
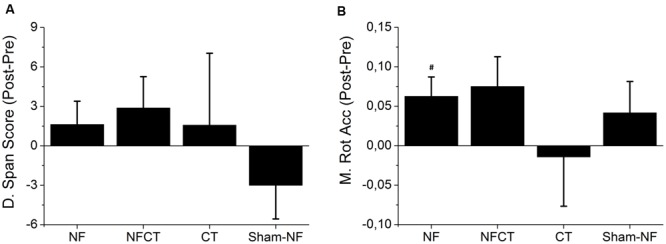
**Representation of the mean difference between pre- and post-training performance in the cognitive tests. (A)** Digit Span Test Score and **(B)** Matrix Rotation Test Accuracy, for all experimental groups: NF (*N* = 8), NFCT (*N* = 8), CT (*N* = 7) and Sham-NF (*N* = 6). NF group presented a statistically significant increase in Matrix Rotation Accuracy (*p*-value = 0.039). ^#^*p* < 0.05.

**Figure 3** represents the differences in performance between pre- and post-intervention for all experimental groups in Digit Span Test and Matrix Rotation Test. In the Digit Span Test, NF, NFCT and CT groups tended to increase the score when comparing pre- and post-intervention but these increases were not statistically significant (NF: *p*-value = 0.219; NFCT: *p*-value = 0.191; CT: *p*-value = 0.500). In the Matrix Rotation Test all groups but the CT tended to increase accuracy (NFCT: *p*-value = 0.063; Sham-NF: *p*-value = 0.25); however, only NF participants significantly increased their performance (*p*-value = 0.039).

**FIGURE 4:**
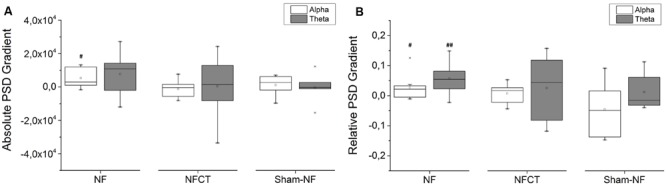
**Representation of the 4-day variation of alpha and theta gradients during training; (A)** absolute PSD and **(B)** relative PSD, for NF (*N* = 9), NFCT (*N* = 8) and Sham-NF (*N* = 6) groups. NF group presented a statistically significant increase in absolute alpha (*p*-value = 0.014) and in relative alpha and theta (*p*-value = 0.027 and 0.006, respectively). ^#^*p* < 0.05, ^##^*p* < 0.01.

**Figure 4A** represents the training gradients of absolute PSD values for NF, NFCT and sham-NF groups across the 4 days of alpha and the 4 days of theta training (see EEG signal processing section). The positive values obtained in the NF group indicate that this group was able to increase both frequency bands activity during NF training. Only alpha band activity increased significantly (*p*-value = 0.014), whereas theta band activity did not, although close to significance (*p*-value = 0.064). Interestingly, the NFCT group did not show any important tendency and the Sham-NF group did not achieve significant changes. **Figure 4B** represents the training gradients of relative PSD values during training. These results indicate that the relative power of alpha and theta bands was significantly increased in the NF group (*p*-value = 0.027 and 0.006 respectively), but not in NFCT or sham-NF groups.

**FIGURE 5:**
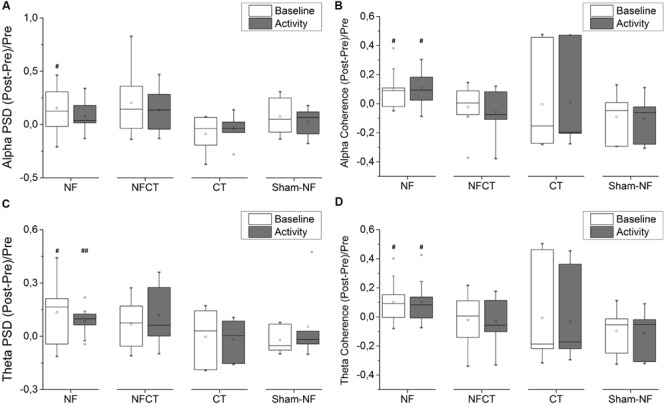
**Evolution of frontal EEG power spectral density (PSD) and frontal spectral coherence from pre- to post-training phase; (A)** alpha and **(C)** theta PSD, on baseline and activity periods, in Fz channel, for NF (*N* = 9), NFCT (*N* = 8), CT (*N* = 7) and Sham-NF (*N* = 6) groups; NF group presented a statistically significant increase in basline alpha (*p*-value = 0.049) and both baseline and activity theta (*p*-value = 0.037 and 0.010, respectively, ^#^*p* < 0.05, ^##^*p* < 0.01); for **(B)** alpha and **(D)** theta frontal spectral coherence (between FL and FR channel pools), for all four experimental groups. NF group presented a statistically significant increase in both baseline and activity alpha (*p*-value = 0.037 and 0.027, respectively) and in both baseline and activity theta (*p*-value = 0.049 and 0.049, respectively, ^#^*p* < 0.05).

**Figure 5** represent the results obtained from the EEG acquired before and after the 8 days of training. **Figures 5A,C** represent relative PSD measured in the Fz electrode in alpha and theta bands, respectively. Only the NF group could increase significantly the PSD in both theta (during baseline – *p*-value = 0.037 and activity – *p*-value = 0.010) and alpha (during baseline – *p*-value = 0.049) rhythms. Although not statistically significant, NFCT presented a tendency to increased relative PSD values in theta (during baseline – *p*-value = 0.125 and activity – *p*-value = 0.055) and alpha (during baseline – *p*-value = 0.074 and activity – *p*-value = 0.098) rhythms. No statistically significant results were obtained using the absolute PSD measures. **Figures 5B,D** represent coherence measured between frontal sites (frontal left – FL and frontal right – FR) in alpha and theta bands, respectively. Again, only the NF group was able to significantly increase alpha and theta coherence in baseline (*p*-value = 0.037 and 0.049, alpha and theta respectively) and activity (*p*-value = 0.027 and 0.049, alpha and theta respectively) records.

**FIGURE 6:**
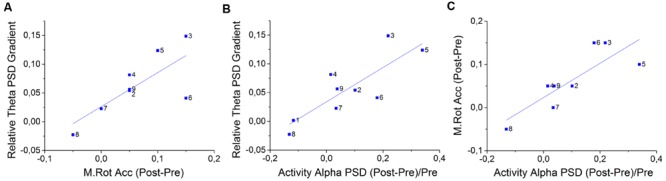
**Correlations between the Fz relative theta gradients, accuracy gains on the Matrix Rotation Test and improvements of post-training Fz alpha PSD for all NF group’s participants (*N* = 8).** Specifically, the positive correlations between **(A)** relative theta gradient and M. Rot. accuracy (Kendall′s τ = 0.617, *p*-value = 0.040) **(B)** relative theta gradient and post-training alpha PSD during activity periods (τ = 0.556, *p*-value = 0.037) and **(C)** M. Rot. accuracy gains and post-training alpha PSD (τ = 0.694, *p*-value = 0.021); Additionally, there is a non-represented positive correlation between the relative theta training gradient and score gains in the D. Span task (τ = 0.718, *p*-value = 0.016); In-plot dots represent participants.

**Figure 6A** represents the positive correlation found between theta gradient during NF training and Matrix Rotation accuracy differences between pre and post NF intervention (Correlation Coef. = 0.617, *p*-value = 0.040). **Figure 6B** represents the positive correlation between theta gradient and alpha PSD differences between pre and post NF intervention (Correlation Coef. = 0.556, *p*-value = 0.037). **Figure 6C** represents the positive correlation between this alpha PSD and the above mentioned matrix rotation accuracy, both measures representing differences between pre and post NF intervention (Correlation Coef. = 0.694, *p*-value = 0.021). Additionally, a positive correlation was found between the theta gradient and the difference between pre- and post-training in the Digit Span score (Correlation Coef. = 0.718, *p*-value = 0.016; not shown). Reliability analyses were applied on all three measures (i.e., relative theta PSD gradient, alpha PSD and Matrix Rotation accuracy) analyzed on **Figure 6**, and the Cronbach’s α resulted in 0.823 (standardized α = 0.922) when only NF subjects were considered. The Cronbach’s α analyzed exclusively for NFCT and Sham-NF subjects resulted in 0.509 and 0.297, respectively. These results suggest that the participants that were able to better modulate theta across the 4 days of training uniquely increase their WM performance, translated in an increase in accuracy in the matrix rotation test (**Figure 6A**) and in score in Digit Span from the pre- to the post-intervention moments. Besides, we also found that only these participants enhanced their background alpha activity in Fz location from pre- to post-training periods (**Figure 6B**). Predictably, the subjects with highest accuracy gains in Matrix Rotation also obtained highest improvements in background alpha PSD (**Figure 6C**).

**FIGURE 7:**
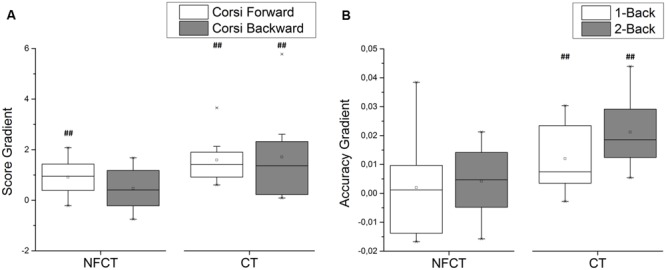
**Cognitive training performance for NFCT and CT groups. (A)** The participants from CT experimental group have shown performance gains during both forward (*p*-value: 0.004) and backward (*p*-value: 0.004) Corsi tasks as well as during 1-back (*p*-value: 0.007) and 2-back (*p*-value: 0.004) tasks. **(B)** The participants from NFCT group only presented performance gains for the forward version of Corsi task (*p*-value: 0.006). CT participants over performed NFCT participants in the 2-back task (*p*-value: 0.016).

**Figure 7** presents the results of cognitive training performance from the participants of NFCT and CT groups. The participants from CT group have shown performance gains during both forward (*p*-value = 0.004) and backward (*p*-value = 0.004) Corsi tasks as well as during 1-back (*p*-value = 0.007) and 2-back (*p*-value = 0.004) tasks. The participants from NFCT group only presented performance gains for the forward version of Corsi task (*p*-value = 0.006). CT participants over performed NFCT participants in the 2-back task (*p*-value = 0.016).

## Discussion

The goal of this study was to assess the effects of an intensive alpha and theta NF protocol in WM performance in a healthy population above 55 years old, as well as to initially explore the benefits of a multimodal cognitive training approach, by supplementing NF protocols with traditional cognitive tasks. Importantly, these subjects were cognitively assessed before the intervention, using traditional neuropsychological scales (MMSE, MOCA, D.Direct, FAS, Codif., and CLOX) and the computerized versions of Matrix Rotation and Digit Span Tests; the results (**Table 1**) show statistically significant correlations between the tests, which provides confidence about the behavioral results of the adapted computerized tests used for WM assessment.

The NF group showed improvements in the Digit Span and Matrix Rotation performance after eight consecutive days of training (**Figure 3**), but only the latter presents statistically significant differences. Together with the fact that the NF group was uniquely able to modulate both alpha and theta frequency bands in respect to the broadband spectrum (**Figure 4**), it seems that a specific up-modulation of alpha or theta activity may have had positive effects in cognitive performance. This finding comes in line with other studies that have already shown the relationship between a successful upper-alpha training and mental rotation enhancement (Hanslmayr et al., 2005) and alpha training and a better performance in a Matrix Rotation task (Riečanský and Katina, 2010). The participants that underwent NF training supplemented with cognitive training (i.e., NFCT group) presented a tendency to increase performance in Matrix Rotation Accuracy (**Figure 3**). Although these results may suggest a potential advantage of a combined cognitive training approach, their failure to show significance is likely due to the reduced dosage of NF training blocks in comparison to the NF group. In fact, the NFCT group only performed 3 5-min blocks of NF in each training session in order to maintain the total training time of 30 min per day. That amount of NF training seems insufficient to fully promote NF learning in all participants. In addition, the implemented cognitive training tasks *per se* seemed to have failed in endorsing cognitive enhancement. Indeed, the CT group did not increase the performance in matrix rotation or digit span scores after the 8 days of intensive cognitive training.

The absolute PSD gradients across the 4 days of alpha and 4 days of theta training (**Figure 4**) show that the NF group presented a tendency to increase PSD in both alpha and theta rhythms but the increase was only statistically significant in alpha. Sham-NF subjects, although not receiving a real feedback of their own EEG signals, also tended to increase the absolute value of alpha power, but not theta power. This could be explained by the nature of the protocol itself and the engagement of attention processes that are often associated with the alpha rhythm. As the subjects must pay attention to the visual stimulus presented in the computer screen, it is possible that even the sham-NF participants had increase alpha rhythm even without receiving the real feedback of their own EEG signals. Also, alpha was reported as being a dominant rhythm in the adult EEG (Klimesch, 1999) and this could explain why its enhancement may be easier, especially when compared to theta which is an EEG pattern more common in sleepy stages or cognitive demanding tasks (Vaitl et al., 2005). Interestingly, the NFCT group presents a theta PSD gradient highly variable across participants, which may be also due to insufficient NF training dosage.

The relative PSD gradients across the 4 days of alpha and 4 days theta training show that only the NF group presented an increased PSD in both frequency bands. Given that the relative alpha and theta are calculated as a ratio of the PSD on a frequency band to the broadband PSD, the increase in relative PSD could be explained by an energy specificity improvement in the underline alpha and theta bands during NF training. In contrast, the Sham-NF group did not improve neither alpha nor theta relative bands, showing the inability of unspecific-feedback to effectively modulate EEG rhythms. The issue of specificity is an assumption inherent to NF practice and it contrasts with an alternative view that suggests that only a generalized learning process is trained. Although our results suggest an energy specificity improvement in the NF group, the evidence is mixed for both specific and generalized effects. Taking into account that NF is a complex process that requires attention, motivation and learning effort, that could explain why it could involve different EEG rhythms besides the target ones (Gruzelier, 2014c).

The analysis of the EEG acquired before and after training, during the computerized battery (**Figure 5**), revealed that only the participants from the NF group were able to increase alpha and theta rhythms in the Fz location from pre- to post-intervention. NFCT group showed only a tendency to increase Fz alpha and theta during baseline and activity, which points to the need of a NF protocol with enough sessions to promote learning and alterations in EEG during task and resting states. The analysis of EEG acquired during the pre- and post-intervention also revealed, in the NF group, an improvement in spectral FL-FR coherence in alpha and theta bands during both activity and baseline. Nevertheless, this improvement did not correlate with NF training success or with cognitive performance improvements. Topological specificity in NF has little support in the literature. However, studies with elder populations provided evidence for frontal locus changes following NF training (Angelakis et al., 2004; Becerra et al., 2012), as a result of the higher engagement of frontal lobes in executive functions and learning. However, in the view of the brain as a group of functional system networks it should be expected that the effects of EEG NF training might be generalized and not restricted to the region of training (Becerra et al., 2012; Gruzelier, 2014b).

The positive correlation between a successful theta NF training and a better performance in matrix rotation task in the NF group, as well as an increased alpha activity between pre- and post-training in these subjects (**Figure 6**), were relevant findings of the present study. The results of these three measures, together with their internal consistency in the NF group, seems to indicate that a successful theta NF training following the proposed protocol could have led to the improvement of alpha EEG and performance in a task involving WM during a computerized battery. These results could indicate that the promoted changes in the alpha PSD could have been triggered by a successful theta up-training and might have enabled a better performance in matrix rotation task, which involves short-term memory and attention abilities. However, these results should be carefully analyzed, since these are correlations, and additionally no statistical significance was found between different groups.

Regarding cognitive training, only the CT group was able to follow cognitive training and improve performance on tasks (**Figure 7**). Again, the failure of NFCT participants to follow training may be due to the reduced dosage in comparison to the CT group. Yet, and although the participants from CT group were able to achieve highest score gradients on Corsi and n-Back tasks, those accomplishments did not translate into either better cognitive performance or post-pre EEG effects. On the other hand, NFCT participants did not show evidence of cognitive enhancement during training but they tended to increase PSD from previous to posterior training moments. These results might suggest that evident training improvement might not be a requirement for achieving effective cognitive gains.

The results presented herein suggest that an intensive no-interval NF training protocol may be adequate for an appropriate learning of EEG modulation. This protocol triggered some alterations in basal EEG that seem to support a better performance in tasks involving short-term memory and attention skills. This is in line, with empirical evidence presented in the literature that seems to indicate that an 8–10-session intervention might be sufficient for young healthy participants (Angelakis et al., 2007); herein, we show that the same applies to elder subjects (Lecomte, 2011). Although positive results have been herein shown for elders, more training sessions might be advisable for an aged population in order to promote enduring effects. The explanation could be the reduced neuroplasticity observed in elder individuals (Goh and Park, 2009) thus, impairing the mnesic processes necessary in a learning process.

## Conclusion

In essence, the major conclusions of this study are, firstly, the moderately positive results on EEG modulation during intensive no-interval 8 NF sessions and their outcome in terms of WM in healthy elder subjects. Multimodal cognitive training approach failed to prove efficacy but revealed promising insights to future study designs aiming to combine NF with cognitive tasks. We also conclude, in line with other studies, that age does not exclude elderly people from learning NF.

## Author Contributions

JR and AMP, contributed to all stages of the work including the acquisition, analysis, and interpretation of data for the work, drafting the work and final approval for publishing. LF, NA, and MP contributed for the acquisition and interpretation of data for the work, revising the manuscript for important intellectual content and final approval. NS and NSD are mainly responsible for the conception and design of the work, contributed for the interpretation of data for the work, revised the work critically for important intellectual content and also gave final approval of the version to be published.

## Conflict of Interest Statement

The authors declare that the research was conducted in the absence of any commercial or financial relationships that could be construed as a potential conflict of interest.

## References

[B1] Adrian E. D. Matthews B. H. C. (1934). The berger rhythm: potential changes from the occipital lobes in man. *Brain* 57 355–385. 10.1093/brain/57.4.355 20058345

[B2] Angelakis E. Lubar J. F. Stathopoulou S. (2004). Electroencephalographic peak alpha frequency correlates of cognitive traits. *Neurosci. Lett.* 371 60–63. 10.1016/j.neulet.2004.08.041 15500967

[B3] Angelakis E. Stathopoulou S. Frymiare J. L. Green D. L. Lubar J. F. Kounios J. (2007). EEG neurofeedback: a brief overview and an example of peak alpha frequency training for cognitive enhancement in the elderly. *Clin. Neuropsychol.* 21 110–129. 10.1080/13854040600744839 17366280

[B4] Babiloni C. Squitti R. Del Percio C. Cassetta E. Ventriglia M. C. Ferreri F. (2007). Free copper and resting temporal EEG rhythms correlate across healthy, mild cognitive impairment, and Alzheimer’s disease subjects. *Clin. Neurophysiol.* 118 1244–1260. 10.1016/j.clinph.2007.03.016 17462944

[B5] Balota D. A. Dolan P. O. Duchek J. M. (2000). “Memory changes in healthy older adults,” in *The Oxford handbook of memory* eds Tulving E. Craik F. I. M. (New York, NY: Oxford University Press) 395–409.

[B6] Bauer R. H. (1976). Short-term memory: EEG alpha correlates and the effect of increased alpha. *Behav. Biol.* 17 425–433. 10.1016/S0091-6773(76)90793-8 971195

[B7] Becerra J. Fernández T. Roca-Stappung M. Díaz-Comas L. Galán L. Bosch J. (2012). Neurofeedback in healthy elderly human subjects with electroencephalographic risk for cognitive disorder. *J. Alzheimers Dis.* 28 357–367. 10.3233/JAD-2011-111055 22002790

[B8] Breslau J. Starr A. Sicotte N. Higa J. Buchsbaum M. S. (1989). Topographic EEG changes with normal aging and SDAT. *Electroencephalogr. Clin. Neurophysiol.* 72 281–289. 10.1016/0013-4694(89)90063-1 2467793

[B9] Cantero J. L. Atienza M. Stickgold R. Kahana M. J. Madsen J. R. Kocsis B. (2003). Sleep-dependent theta oscillations in the human hippocampus and neocortex. *J. Neurosci.* 23 10897–10903. 14645485 10.1523/JNEUROSCI.23-34-10897.2003 PMC6740994

[B10] Cavanagh J. F. Zambrano-Vazquez L. Allen J. J. B. (2012). Theta lingua franca: a common mid-frontal substrate for action monitoring processes. *Psychophysiology* 49 220–238. 10.1111/j.1469-8986.2011.01293.x 22091878 PMC3262926

[B11] Cepeda N. J. Kramer A. F. Gonzalez de Sather J. C. (2001). Changes in executive control across the life span: examination of task-switching performance. *Dev. Psychol.* 37 715–730. 10.1037/0012-1649.37.5.715 11552766

[B12] Coben R. Linden M. Myers T. E. (2010). Neurofeedback for autistic spectrum disorder: a review of the literature. *Appl. Psychophysiol. Biofeedback.* 35 83–105. 10.1007/s10484-009-9117-y 19856096

[B13] Connelly S. L. Hasher L. Zacks R. T. (1991). Age and reading: the impact of distraction. *Psychol. Aging.* 6 533–541. 10.1037/0882-7974.6.4.533 1777141

[B14] Delorme A. Makeig S. (2004). EEGLAB: an open source toolbox for analysis of single-trial EEG dynamics including independent component analysis. *J. Neurosci. Methods* 134 9–21. 10.1016/j.jneumeth.2003.10.009 15102499

[B15] Dias N. S. Ferreira D. Reis J. Jacinto L. R. Fernandes L. Pinho F. (2015). Age effects on EEG correlates of the Wisconsin card sorting test. *Physiol. Rep.* 3 e12390 10.14814/phy2.12390 PMC4552514 26216431

[B16] Egner T. Sterman M. B. (2006). Neurofeedback treatment of epilepsy: from basic rationale to practical application. *Expert Rev. Neurother.* 6 247–257. 10.1586/14737175.6.2.247 16466304

[B17] Enriquez-Geppert S. Huster R. J. Scharfenort R. Mokom Z. N. Zimmermann J. Herrmann C. S. (2014). Modulation of frontal-midline theta by neurofeedback. *Biol. Psychol.* 95 59–69. 10.1016/j.biopsycho.2013.02.019 23499994

[B18] Escolano C. Aguilar M. Minguez J. (2011). EEG-based upper alpha neurofeedback training improves working memory performance. *Conf. Proc. IEEE Eng. Med. Biol. Soc.* 2011 2327–2330. 10.1109/IEMBS.2011.6090651 22254807

[B19] Giaquinto S. Nolfe G. (1986). The EEG in the normal elderly: a contribution to the interpretation of aging and dementia. *Electroencephalogr. Clin. Neurophysiol.* 63 540–546. 10.1016/0013-4694(86)90141-0 2422003

[B20] Goh J. O. Park D. C. (2009). Neuroplasticity and cognitive aging: the scaffolding theory of aging and cognition. *Restor. Neurol. Neurosci.* 27 391–403. 10.3233/RNN-2009-0493 19847066 PMC3355626

[B21] Grady C. (2000). Changes in memory processing with age. *Curr. Opin. Neurobiol.* 10 224–231. 10.1016/S0959-4388(00)00073-8 10753795

[B22] Gruzelier J. H. (2014a). EEG-neurofeedback for optimising performance. I: a review of cognitive and affective outcome in healthy participants. *Neurosci. Biobehav. Rev.* 44 124–141. 10.1016/j.neubiorev.2013.09.015 24125857

[B23] Gruzelier J. H. (2014b). EEG-neurofeedback for optimising performance. II: creativity, the performing arts and ecological validity. *Neurosci. Biobehav. Rev.* 44 142–158. 10.1016/j.neubiorev.2013.11.004 24239853

[B24] Gruzelier J. H. (2014c). EEG-neurofeedback for optimising performance. III: a review of methodological and theoretical considerations. *Neurosci. Biobehav. Rev.* 44 159–182. 24690579 10.1016/j.neubiorev.2014.03.015

[B25] Hammond D. C. (2005). Neurofeedback treatment of depression and anxiety. *J. Adult Dev.* 12 131–137. 10.1007/s10804-005-7029-5

[B26] Hammond D. C. (2011). What is Neurofeedback: an Update. *J. Neurother.* 15 305–336. 10.1080/10874208.2011.623090

[B27] Hanslmayr S. Sauseng P. Doppelmayr M. Schabus M. Klimesch W. (2005). Increasing individual upper alpha power by neurofeedback improves cognitive performance in human subjects. *Appl. Psychophysiol. Biofeedback* 30 1–10. 10.1007/s10484-005-2169-8 15889581

[B28] Heinrich H. Gevensleben H. Strehl U. (2007). Annotation: neurofeedback – train your brain to train behaviour. *J. Child Psychol. Psychiatry* 48 3–16. 10.1111/j.1469-7610.2006.01665.x 17244266

[B29] Itthipuripat S. Wessel J. R. Aron A. R. (2013). Frontal theta is a signature of successful working memory manipulation. *Exp. Brain Res.* 224 255–262. 10.1007/s00221-012-3305-3 23109082 PMC3536917

[B30] Jacobs J. Hwang G. Curran T. Kahana M. J. (2006). EEG oscillations and recognition memory: theta correlates of memory retrieval and decision making. *Neuroimage.* 32 978–987. 10.1016/j.neuroimage.2006.02.018 16843012

[B31] Klimesch W. (1997). EEG-alpha rhythms and memory processes. *Int. J. Psychophysiol.* 26 319–340. 10.1016/S0167-8760(97)00773-3 9203012

[B32] Klimesch W. (1999). EEG alpha and theta oscillations reflect cognitive and memory performance: a review and analysis. *Brain Res. Rev.* 29 169–195. 10.1016/S0165-0173(98)00056-3 10209231

[B33] Kramer A. F. Humphrey D. G. Larish J. F. Logan G. D. Strayer D. L. (1994). Aging and inhibition: beyond a unitary view of inhibitory processing in attention. *Psychol. Aging* 9 491–512. 10.1037/0882-7974.9.4.491 7893421

[B34] Lecomte G. (2011). The effects of neurofeedback training on memory performance in Elderly subjects. *Psychology.* 2 846–852. 10.4236/psych.2011.28129

[B35] Lustig C. Shah P. Seidler R. Reuter-Lorentz P. A. (2009). Aging, training and the brain: a review and future directions. *Dialog. Clin. Neurosci.* 15 109–119. 10.1007/s11065-009-9119-9 PMC3005345 19876740

[B36] Madden D. J. (1990). Adult age differences in the time course of visual attention. *J. Gerontol.* 45 9–16. 10.1093/geronj/45.1.P9 2295780

[B37] Makeig S. J. Bell A. Jung T.-P. Sejnowski T. J. (1996). Independent component analysis of electroencephalographic data. *Adv. Neural. Inf. Process. Syst.* 8 145–151.

[B38] Mueller S. T. Piper B. J. (2014). The psychology experiment building language (PEBL) and PEBL test battery. *J. Neurosci. Methods* 222 250–259. 10.1016/j.jneumeth.2013.10.024 24269254 PMC3897935

[B39] Perego P. Maggi L. Parini S. (2009). “BCI ++: a New framework for brain computer interface application,” In *Proceedings of the 18th International Conference on Software Engineering and Data Engineering* Las Vegas, NY 37–41.

[B40] Prichep L. S. (2007). Quantitative EEG and electromagnetic brain imaging in aging and in the evolution of dementia. *Ann. N. Y. Acad. Sci.* 1097 156–167. 10.1196/annals.1379.008 17413018

[B41] Renard Y. Lotte F. Gibert G. Congedo M. Maby E. Delannoy V. (2010). OpenViBE: an open-source software platform to design, test, and use brain–computer interfaces in real and virtual environments. *Presence.* 19 35–53. 10.1162/pres.19.1.35

[B42] Riečanský I. Katina S. (2010). Induced EEG alpha oscillations are related to mental rotation ability: the evidence for neural efficiency and serial processing. *Neurosci. Lett.* 482 133–136. 10.1016/j.neulet.2010.07.017 20637833

[B43] Salthouse T. A. (1996). The processing-speed theory of adult age differences in cognition. *Psychol. Rev.* 103 403–428. 10.1037/0033-295X.103.3.403 8759042

[B44] Staufenbiel S. M. Brouwer A.-M. Keizer A. W. van Wouwe N. C. (2014). Effect of beta and gamma neurofeedback on memory and intelligence in the elderly. *Biol. Psychol.* 95 74–85. 10.1016/j.biopsycho.2013.05.020 23751914

[B45] Vaitl D. Birbaumer N. Gruzelier J. Jamieson G. A. Kotchoubey B. Kübler A. (2005). Psychobiology of altered states of consciousness. *Psychol. Bull.* 131 98–127. 10.1037/0033-2909.131.1.98 15631555

[B46] Vernon D. J. (2005). Can neurofeedback training enhance performance? An evaluation of the evidence with implications for future research. *Appl. Psychophysiol. Biofeedback* 30 347–364. 10.1007/s10484-005-8421-4 16385423

[B47] Wang J.-R. Hsieh S. (2013). Neurofeedback training improves attention and working memory performance. *Clin. Neurophysiol.* 124 2406–2420. 10.1016/j.clinph.2013.05.020 23827814

[B48] Zoefel B. Huster R. J. Herrmann C. S. (2011). Neurofeedback training of the upper alpha frequency band in EEG improves cognitive performance. *Neuroimage* 54 1427–1431. 10.1016/j.neuroimage.2010.08.078 20850552

